# Role of Glial Immunity in Lifespan Determination: A *Drosophila* Perspective

**DOI:** 10.3389/fimmu.2018.01362

**Published:** 2018-06-11

**Authors:** Ilias Kounatidis, Stanislava Chtarbanova

**Affiliations:** ^1^Cell Biology, Development, and Genetics Laboratory, Department of Biochemistry, University of Oxford, Oxford, United Kingdom; ^2^Department of Biological Sciences, University of Alabama, Tuscaloosa, AL, United States

**Keywords:** *Drosophila*, innate immunity, glia, lifespan, neurodegeneration, phagocytosis

## Abstract

Increasing body of evidence indicates that proper glial function plays an important role in neuroprotection and in organismal physiology throughout lifespan. Work done in the model organism *Drosophila melanogaster* has revealed important aspects of glial cell biology in the contexts of longevity and neurodegeneration. In this mini review, we summarize recent findings from work done in the fruit fly *Drosophila* about the role of glia in maintaining a healthy status during animal’s life and discuss the involvement of glial innate immune pathways in lifespan and neurodegeneration. Overactive nuclear factor kappa B (NF-κB) pathways and defective phagocytosis appear to be major contributors to lifespan shortening and neuropathology. Glial NF-κB silencing on the other hand, extends lifespan possibly through an immune–neuroendocrine axis. Given the evolutionary conservation of NF-κB innate immune signaling and of macrophage ontogeny across fruit flies, rodents, and humans, the above observations in glia could potentially support efforts for therapeutic interventions targeting to ameliorate age-related pathologies.

## Introduction

Organismal aging is a complex phenomenon resulting in the progressive decline of physiological functions and increased susceptibility to death (1). Both, genetic and environmental factors are believed to contribute to the aging process and lifespan (1–3). Work in several model organisms including the invertebrates *Drosophila melanogaster* and *Caenorhabditis elegans* have identified genes and cellular pathways conserved through evolution that affect longevity such as the insulin-like pathway or the target of rapamycin (TOR) pathway (4, 5). Thus, these model organisms proved to be of valuable use for studying the molecular mechanisms that underlie aging.

*Drosophila*, the common fruit fly, is an excellent versatile model organism to investigate the interplay between innate immune function and brain physiology among the effects of this interaction to host lifespan. There is a high degree of evolutionary conservation of the molecular mechanisms of innate immunity between flies and mammals. For instance, the two *Drosophila* nuclear factor kappa B (NF-κB)-based pathways, namely Toll and Immune deficiency (IMD) share similarities with the mammalian Toll-like receptor pathways and tumor necrosis factor receptor 1 pathways, respectively (6–10). In the context of bacterial and fungal infections, activation of these pathways leads to the translocation of NF-κB factors (Relish for IMD and Dif and Dorsal for Toll pathway, respectively) from the cytoplasm into the nucleus of the cell allowing transcription and synthesis of potent antimicrobial peptides (AMPs) (10). Phagocytosis is another powerful mechanism to eliminate cellular debris or infection that has been conserved during evolution (11, 12). In mammals, phagocytosis is mediated by cell surface receptors, which bind bacteria or apoptotic bodies either directly or via opsonins (13). In flies, several phagocytic recognition receptors have been identified on hemocytes (the fly macrophage-like cells), among which is the EGF-like repeat-containing protein Draper (12). Draper has also been implicated in the removal of apoptotic neurons during *Drosophila* nervous system development (14) and metamorphosis (15) as well as in phagocytosis of axonal debris after axonal injury (16–18). Flies have also significantly contributed to advances in studies of neurodegeneration such as the identification of novel neuroprotective genes and provided information about conserved processes required for maintaining the structural integrity of the central nervous system (CNS) (19). Moreover, several human neurodegenerative diseases such as Alzheimer’s, Parkinson’s, and Huntington’s disease have been effectively modeled in *Drosophila* yielding insights into the molecular base of these disorders (20).

The chronic inflammatory status that accompanies human aging, also known as inflammaging, is considered a significant risk factor for many chronic pathologies including cancer, cardiovascular and neurodegenerative disorders (21). In the context of aging, increased levels of pro-inflammatory cytokines such as TNF-alpha and Interleukine (IL)-6 are found upregulated in brain tissue (22). With age, mammalian microglia, which are the brain immune cells exhibit primed profile characterized by increased activation and enhanced secretion of pro-inflammatory cytokines (23, 24). Decline in microglial function, migration and chemotaxis are also observed with age (24). For instance, microglia’s engulfment capacity of amyloid-beta (Aβ) (25) or alpha-synuclein (α-Syn) (26) oligomers, whose accumulation is characteristic for Alzheimer’s and Parkinson’s disease, respectively, are compromised in aged animals. Moreover, activated microglia and neuroinflammatory profiles are observed in most neurodegenerative disorders including Huntington’s (27), Alzheimer’s (28, 29), and Parkinson’s (30–32) diseases and are believed to underlie the onset, severity, and progression of these disorders (24). Similar to mammalian models, both chronic innate immune activation (4, 33) as well as decline in phagocytic activity of glia (18) are observed in the aging *Drosophila* brain. It is thus apparent that glial immunity is linked to both, healthy aging and age-dependent neurodegeneration. In the mammalian brain, under normal physiological conditions, microglia provide the first line of defense against brain injury and infection. These cells are able to sense pathogens *via* pathogen recognition receptors, activate innate immune signaling pathways, phagocytose microorganisms, and clear cellular debris (34). Microglia also have the capacity to secrete neurotrophic factors and anti-inflammatory molecules, therefore, playing a protective role in these contexts. On the other hand, the neurodegenerative process itself can trigger inflammation (34–36), leading to detrimental effects on the brain. It is, therefore, important to understand the mechanisms by which, changes in the same signaling pathway (e.g., NF-kB) lead to two distinct phenotypes, namely healthy aging associated with neuroprotection and neurodegeneration.

Glial cells are essential players in CNS development and in maintaining homeostasis in this tissue (37). Glial cells provide trophic support to neurons, regulate ionic homeostasis in the brain, and serve as immune cells that are armed to respond to injury or infection (37). Increasing body of evidence indicates that dysfunction of diverse cellular processes specifically in glial cells may have profound impact on animal’s survival and, therefore, affect life expectancy. We review here recent discoveries of the role played by glial cells in animal’s lifespan, as well as how glial innate immune pathways relate to organismal longevity in the model organism *Drosophila melanogaster*.

## Glial Types and their Contribution to Healthy Aging

Glial cells play major roles in nervous system development, synapse formation, plasticity, and brain homeostasis (38, 39). Five major morphologically distinct classes of glial cells with diverse functions can be appreciated in the brain of adult *Drosophila* (38–40) and additional glial subtypes in its visual system (40, 41). Among brain glia, perineurial and subperineurial glia form the blood–brain barrier (BBB) to isolate the brain from the potassium-rich hemolymph (insect blood) assuring optimal neuronal function (42–44). To meet the high-energy demands of neurons, glia supply neurons with metabolites through an evolutionary conserved process known as metabolic coupling (45). *Drosophila* BBB glia support neurons by providing them metabolic factors derived from the break down of the sugar trehalose (45). Glia-, but not neuron-specific silencing of the genes encoding the glycolytic enzymes *Trehalase* and *Pyruvate kinase* substantially shortens flies’ lifespan and leads to neurodegeneration (45). Interestingly, glia-specific knock down of another gene encoding an enzyme involved in glycolysis, *Aldolase*, leads to neurodegeneration and shortened lifespan (46). Together, these studies indicate that glial glycolysis plays an important role in healthy aging and neuroprotection. Along the same lines, mutations in the gene encoding the glia-enriched monocarboxylate transporter *Chaski*, lead to shortened lifespan, synaptic dysfunction, and locomotor impairment pointing to an important role for glial transport of metabolites during the animal’s lifespan (47). This work is of particular importance as it is becoming increasingly evident that metabolic changes, among which lower brain glucose metabolism, accompanies aging and Alzheimer’s disease in humans (48). Metabolomics analysis of mouse brain samples reveals compromised energy state in the aging brain, possibly affecting glial cells that supply glycolytic substrates to neurons (49).

Ensheathing glia, which express the engulfment receptor Draper are the main subtype of adult *Drosophila* glial cells that phagocytose axonal debris following nerve injury (50). Cortex glia play an important trophic role for neurons in the adult brain (38) and are important for neuronal excitability as downregulation of a potassium-dependent sodium/calcium exchanger in this glial subtype leads to seizures in the adult (51). Astrocytes, which share morphological and functional properties with mammalian astrocytes (38) are major contributors to the maintenance of neurotransmitter homeostasis and are involved in regulating circadian rhythmicity in the adult (52). In the *Drosophila*, adult visual system several glial subtypes among, which epithelial glia play a role in synaptic transmission and the processing of visual information (41). It was recently reported that lipid droplet accumulation in these glia due to mitochondrial dysfunction in neurons contributes to neurodegeneration (53). The recently discovered Semper glia, which share functional features with mammalian visual system glial cells such as Müller glia, astrocytes, and oligodendrocytes provide support to photoreceptors and prevent light-induced retinal degeneration (40).

Another, unique, microglia-like glial subtype has been recently discovered in *Drosophila*. These cells, called MANF immunoreactive cells (MiCs) are transiently present in the metamorphosing pupal brain, but not in the adult stage, and upon certain conditions in glia, including (*i)* genetic silencing of dmMANF (Mesencephalic astrocyte-derived neurotrophic factor), (*ii)* induction of autophagy via overexpression of *Atg-1* or the dominant-negative form of Target of rapamycin (*TOR^TED^*), and (*iii)* challenge of innate immunity by ectopic expression of IMD-pathway receptors *PGRP-LC* or *PGRP-LE* (54). Although MiCs have features reminiscent of mammalian microglia, they were not observed in brains of 10-day-old fly mutants that exhibit neurodegeneration such as *ATM^8^* (discussed later in the text) and *swiss cheese* or of *Drosophila* α-Syn model of Parkinson’s disease. It appears that MiCs are immunoreactive; they express the NF-κB transcription factor Relish in their nucleus as well as the phagocytic receptor Draper on their surface (54). Interestingly, glia-specific silencing of *dmMANF* also leads to neurodegeneration and shorter lifespan in the adult suggesting a homeostatic role for this gene in glia (55). How exactly MiCs contribute to this phenotype is not known. More studies are needed in order to fully characterize this intriguing glial cell population and how exactly they relate to brain immune function and healthy lifespan. Glial subtypes and their function in the adult are presented in Table 1.

**Table 1:** Glial subtypes and their location and functions in the adult.

Glial subtype	Function in adult	Location	Reference
Cortex glia	– Trophic support to neurons – Regulation of seizure susceptibility	– Brain cortex – Wrap neuronal cell bodies and processes	Kremer et al. (39) Stork et al. (56) Melom et al. (51)

Astrocyte-like glia	– Maintenance of neurotransmitter homeostasis – Circadian rhythm regulation	– Brain neuropil	Kremer et al. (39) Rival et al. (57) Stork et al. (58) Suh et al. (59) Ng et al. (52)

Ensheathing glia	– Phagocytosis of debris after injury – Regulation of olfactory circuit plasticity	– Brain neuropil – Associated with axon tracts	Kremer et al. (39) Doherty et al. (50) Kazama et al. (60)

Perineurial glia	– Blood–brain barrier (BBB) formation and chemoisolaion – Sugar import into the CNS	– Brain surface	Kremer et al. (39) Featherstone (44) Hindle et al. (43) Miller et al. (46) Volkenhoff et al. (45)

Subperineurial glia	– BBB formation and chemoisolaion	– Brain surface	Kremer et al. (39) Featherstone (44) Hindle et al. (43)

MANF immunoreactive cells	– Microglia-like cells	– Pupal brain neuropil	Stratoulias et al. (54)

Adult visual system glia	– Role in synaptic transmission – Prevent light-induced retinal degeneration	– Optic lobe	Chotard et al. (41) Charlton-Perkins et al. (40)

## Role of Glial Immunity in Neurodegeneration and Shortened Lifespan

Prolonged activation of inflammatory responses often translates into harmful consequences ultimately leading to reduction of animal’s lifespan. In the context of brain aging, persistent IMD pathway activation, as well as defective glial clearance function are associated with neurodegenerative phenotypes. Several studies in *Drosophila* highlight the role of glial innate immune responses (phagocytosis as well as NF-κB activation) in promoting neurodegeneration and shortening lifespan. One of the first reports correlating glial activation of the NF-κB ortholog Relish with neurodegenerative phenotypes and lifespan shortening is a study done in a fly model of Ataxia-telangiectasia (A-T) (61). This work shows that glial cells in the fly brain are responsible for increased innate immune activation when ATM kinase activity is reduced. AMPs, which are direct NF-κB transcriptional targets, are up regulated exclusively in glial cells in *ATM^8^* mutants leading to Caspase-3 activation in neighboring neurons suggesting that neurodegeneration could be driven by increase in glial immunity (61). Flies in which *ATM* is specifically silenced in glial cells exhibit shortened lifespan, premature defects in locomotor activity, and spongiform brain pathology in conjunction with activation of Caspase-3 indicative for neurodegeneration (61). Results from a subsequent study done by the same group show that the degree of activation of the innate immune response correlates with the severity of neurodegeneration and lifespan duration in *ATM^8^* mutants and that glial overexpression of a constitutively active form of Relish (Rel-D) leads to neurodegeneration (62). The fact that innate immune activation in brain tissue contributes to neuropathology is supported by findings from other groups that implicate both, activation of the IMD and Toll pathways in *Drosophila* models of light-induced retinal degeneration and Alzheimer’s disease, respectively (63, 64). Pan-neuronal activation of constitutively active Relish results in increased lethality at eclosion pointing to a toxic effect of prolonged immunity in nerve cells (63); however, glial-specific effects of innate immune activation on lifespan and neurodegeneration in these models remains to be determined.

Another piece of evidence for direct implication of the NF-κB-dependent innate immune response in neurodegeneration and longevity comes from the finding that mutation in *defense repressor 1* (*Dnr1*), a negative regulator of the IMD pathway acting at the level of the caspase Dredd, leads to progressive neurodegeneration and reduced lifespan in a Relish-dependent manner (65). In the same study, the authors report that bacterially induced progressive neurodegeneration and resulting locomotor defects are suppressed when *Relish* is specifically knocked down in glial cells. This work goes further by demonstrating that glia-specific overexpression of several AMPs also leads to progressive neurodegeneration. Intriguingly, the overexpression of single AMP-coding genes in glia is sufficient to cause neuropathology and this effect is direct because glial knock down of *Relish* does not suppress *Defensin*- nor *Drosomycin*-induced neurodegeneration (65). A subsequent study demonstrates that glia-specific overexpression of individual AMPs results in impaired locomotor activity and shortened lifespan providing additional evidence for the effect of these NF-κB target genes on fitness and longevity (33).

**Figure 1:**
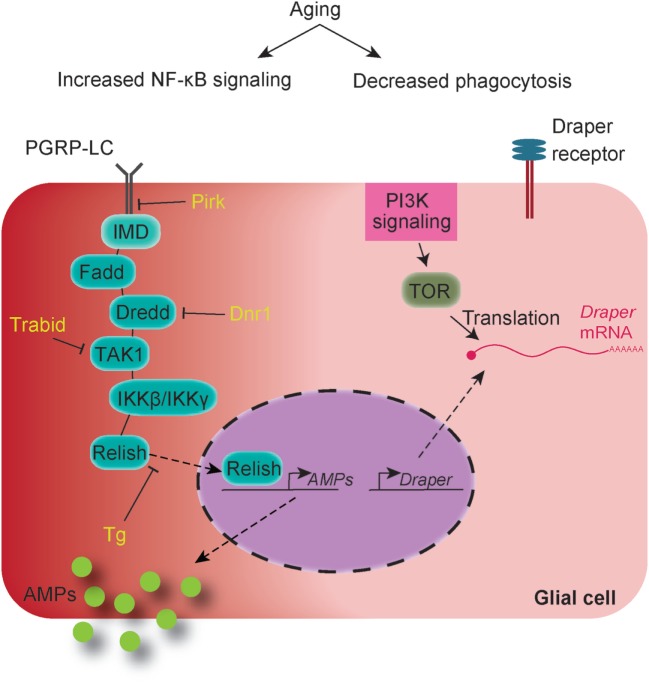
Age-dependent changes in innate immune pathways in *Drosophila* glial cells: immune deficiency (IMD) pathway (on the left) shows age-dependent activation resulting in increased levels of antimicrobial peptides (AMPs) in middle and old-aged adults in absence of microbial challenge. Mutations in genes encoding specific IMD negative regulators namely *Dnr1, trabid, Transglutaminase (Tg,)* and *pirk* release the pathway allowing activation of Relish and subsequent transcription of downstream genes including those encoding AMPs. Aging also affects expression of the phagocytic receptor Draper (on the right) leading to inefficient phagocytic capacity of glial cells. Draper expression levels in the healthy brain are regulated *via* phosphoinositide-3-kinase signaling activity that mediates TOR-dependent translation of *draper* mRNA in glial cells. Age related decline in the activity of this signaling cascade leads to reduction in protein levels of Draper in glia.

The fly IMD pathway is tightly regulated at almost every step of the signaling cascade from the surface to the nucleus of the cell (8, 66). In addition to *Dnr-1*, mutants for other intracellular safeguards of the pathway such as *Pirk, Trabid*, and *Transglutaminase* (*Tg*) that act at the level of the adaptor protein Imd, the kinase TAK1, and the transcription factor Relish, respectively, also exhibit shortened lifespan and neurodegeneration along with brain-specific upregulation of AMPs (33) (Figure 1). Glial silencing of *Relish* in *Trabid* mutants suppresses the age-dependent locomotor impairments and neurodegeneration in these flies and also restores lifespan to almost wild-type levels (33). Altogether, these studies attribute a role for glial NF-κB activation and downstream effectors such as the AMPs in lifespan and neurodegeneration.

Equally important to the overactive NF-κB/Relish branch of the immune response, are the alterations in glial phagocytosis, which are also associated with enhanced neurodegeneration and reduction in lifespan. A recent report showed that while protein levels of the engulfment receptor Draper are reduced in an age-dependent manner, glia’s efficiency in removing cellular debris, such as the ones deriving from degenerating neurons, declines (18). In the context of healthy aging, reduced Draper levels follow an age-associated regression of glial phosphoinositide-3-kinase signaling that mediates TOR-dependent translation of *draper* mRNA (Figure 1) while in situations of neuronal injuries a STAT92E-dependent transcriptional upregulation of *draper* has been described (18, 67). Moreover, flies mutant for Draper exhibit short lifespan (68) and age-dependent neurodegeneration (69), pointing to a neuroprotective role for the phagocytic branch of the innate immune response. Flies, in which *Draper* is silenced specifically in glial cells, also exhibit age-dependent neurodegeneration (69). Persistent apoptotic neurons throughout the lifespan of *Draper* mutants that are not efficiently processed by glial cells due to defects in phagosome maturation appear to be the main reason for the observed phenotypes (69). However, how exactly corpse processing defects cause neurodegeneration remains to be determined.

## Role of Glial Immunity in Lifespan Extension

Kounatidis and colleagues (33) describe an age-dependent shift in IMD-related AMP transcription in *Drosophila* including tissues like the brain of adult flies that is accompanied by neurological impairments such as decreased locomotor performance and increased neurodegeneration in 50-day-old wild-type flies (33). In the same study, the suppression of age-dependent progressive immunity by silencing three components of the IMD pathway, namely *Imd, Dredd*, and *Relish* in glial cells results in increased transcription of the *adipokinetic hormone* (*AKH*)-coding gene concomitant with high nutrient levels later in life and an extension of active lifespan (33). AKH is the fly ortholog of the mammalian gonadotropin-releasing hormone (70), which in mice controls an NF-κB-dependent immune-neuroendocrine axis that is involved in organismal aging (71). A remarkable deceleration of the aging process is recorded in mice upon hypothalamic blocking of NF-κB and the upstream kinase IKK-β (inhibitor of nuclear factor kappa-B kinase subunit beta), followed by an increased median longevity by nearly 20% (71). Similarly, in flies, glial-specific NF-κB immune signaling suppression results in 61% increase in median longevity compared to controls, which is also accompanied by increased locomotor activity in older age (33).

Recent studies in rodents that employ antiaging drugs link lifespan extension with reduction of age-associated overproduction of the pro-inflammatory cytokine TNF-alpha by microglia in both hypothalamus and hippocampus (72). Collectively, these studies implicate glial NF-κB signaling in lifespan determination and point to a role for the immune-neuroendocrine axis in this process.

## Conclusion/Future Directions

It is becoming increasingly evident that glial cells play an important role in neuroprotection and in organismal physiology throughout lifespan. In the recent years, studies in the model organism *Drosophila* have revealed numerous aspects of glial contribution toward both, healthy aging and the development and progression of age-related pathologies of the nervous system. Dysregulation of glial innate immune reactions such as improper NF-κB signaling or impaired Draper-based phagocytosis results in early onset neurodegeneration and lifespan shortening. Thus, both branches of the innate immune response seem to contribute in host neuroprotection and longevity. Additional work is needed to investigate whether these two pieces of the innate immune response possess synergistic properties and identify possible cellular factors that regulate both the inflammatory and phagocytic pathways in glial cells.

Injection of Alzheimer’s disease-related Aβ oligomers (AβOs) into the brains of mice and macaques results in activated pro-inflammatory IKKβ/NF-κB signaling in the hypothalamus and subsequent induction of peripheral glucose intolerance (73). The majority of dementia-related diseases share an inflammation-based branch. Given the evolutionary conservation of innate immune signaling in flies, mice, and humans, strategies like challenging the inflammatory effect of NF-κB pathways could be proved an effective strategy in both “healthy aging” status and in cases of predisposition to age-related neurological diseases.

Additional avenues for future research will involve the studies of the exact mechanisms by which glial effectors downstream of NF-κB such as the AMPs induce neurotoxicity and shorten lifespan. Amyloid-β peptides, which are involved in Alzheimer’s disease pathology have antimicrobial properties (74) and can protect the mouse brain from infection with pathogenic bacteria (75). AMPs can exert bactericidal effects by inserting into and disrupting bacterial membranes (76, 77). Interestingly, modeling studies have suggested that Aβ peptides can also insert into lipid bilayers and potentially form pores in cellular membranes, and thus can damage cells and lead to neurodegeneration (78). Flies can offer an excellent experimental system to address this question and provide important new insights into the mechanisms of AMP toxicity.

Interpolations that boost glial engulfment activity can delay age-dependent processes like delayed clearance of damaged neurons and cellular debris (18). Furthermore, it has been shown that enhanced glial engulfment reverses Aβ accumulation as well as associated behavioral phenotypes in a *Drosophila* AD model (68).

Therefore, glial immune signaling will potentially provide a new cohort of molecular foci for therapeutic interventions in cases of common incurable neurodegenerative disorders in the aging population.

## Author Contributions

IK and SC wrote the manuscript and designed the figures.

## Conflict of Interest Statement

The authors declare that the research was conducted in the absence of any commercial or financial relationships that could be construed as a potential conflict of interest.
